# Clinical characteristics of COVID-19 with cardiac injury: a systematic review and meta-analysis

**DOI:** 10.1017/S0950268820002587

**Published:** 2020-10-23

**Authors:** Linwen Zeng, Shihui Wang, Jianing Cai, Shaoqing Sun, Suhuai Wang, Jingjie Li, Lin Sun

**Affiliations:** Department of Cardiology, The First Affiliated Hospital of Harbin Medical University, Harbin, Heilongjiang, China

**Keywords:** Cardiac injury, coronavirus, COVID-19, meta analysis

## Abstract

**Objectives:**

Cardiac injury is associated with poor prognosis of 2019 novel coronavirus disease 2019 (COVID-19), but the risk factors for cardiac injury have not been fully studied. In this study, we carried out a systematic analysis of clinical characteristics in COVID-19 patients to determine potential risk factors for cardiac injury complicated COVID-19 virus infection.

**Methods:**

We systematically searched relevant literature published in Pubmed, Embase, Europe PMC, CNKI and other databases. All statistical analyses were performed using STATA 16.0.

**Results:**

We analysed 5726 confirmed cases from 17 studies. The results indicated that compared with non-cardiac-injured patients, patients with cardiac injury are older, with a greater proportion of male patients, with higher possibilities of existing comorbidities, with higher risks of clinical complications, need for mechanical ventilation, ICU transfer and mortality. Moreover, C-reactive protein, procalcitonin, D-dimer, NT-proBNP and blood creatinine in patients with cardiac injury are also higher while lymphocyte counts and platelet counts decreased. However, we fortuitously found that patients with cardiac injury did not present higher clinical specificity for chest distress (*P* = 0.304), chest pain (*P* = 0.334), palpitations (*P* = 0.793) and smoking (*P* = 0.234). Similarly, the risk of concomitant arrhythmia (*P* = 0.103) did not increase observably either.

**Conclusion:**

Age, male gender and comorbidities are risk factors for cardiac injury complicated COVID-19 infection. Such patients are susceptible to complications and usually have abnormal results of laboratory tests, leading to poor outcomes. Contrary to common cardiac diseases, cardiac injury complicated COVID-19 infection did not significantly induce chest distress, chest pain, palpitations or arrhythmias. Our study indicates that early prevention should be applied to COVID-19 patients with cardiac injury to reduce adverse outcomes.

## Introduction

**Fig. 1.**
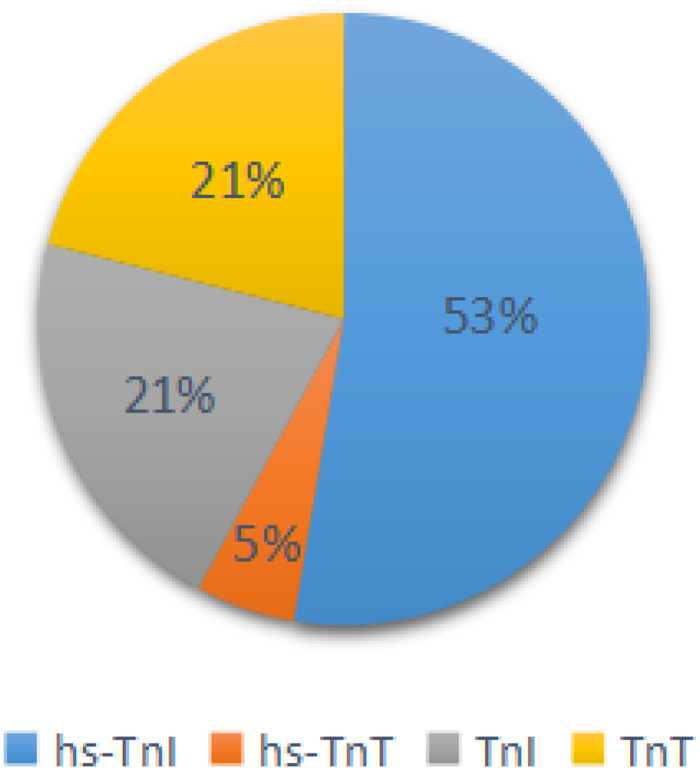
The proportion of articles classified by diagnostic criteria of cardiac injury.

Coronavirus 2019 (COVID-19) caused by severe acute respiratory syndrome coronavirus 2 (SARS-CoV-2) is becoming the primary focus of global health care since December 2019 and there has been 3 267 184 confirmed cases and 229 971 deaths worldwide till 2 May 2020 [1]. According to recent research statistics, the overall fatality rate of COVID-19 is 2.3%, though it is lower than SARS (10%) and MERS (37%), and is markedly increased in patients with cardiovascular disease [2]. Another study revealed that the in-hospital mortality rate of COVID-19 patients with cardiovascular injury reached up to 4.3–28.2% while SARS patients complicated with cardiovascular disease have much lower death rate, around 3.6–13% [3], suggesting that abnormal cardiovascular events may have a profound impact on the disease progression and clinical outcome of COVID-19 patients (Fig. 1).

Cardiac injury is not only the basis of most adverse cardiovascular events such as acute myocardial infarction and myocarditis, but also a prominent characteristic of COVID-19 patients. It is associated with the severity of the disease and the risk of mortality [4, 5]. However, the risk factors for and clinical manifestations of cardiac injury complicated with COVID infection have not been fully studied. About 20–30% of hospitalised patients have a concurrency of cardiac injury and some of them are even more likely to experience cardiac injury as the dominant manifestation rather than fever, cough and other common symptoms of pneumonia [6–8]. This may delay the diagnosis of COVID-19 and increase the risk of spreading virus by those with atypical symptoms. Therefore, in-depth analysis of the clinical characteristics in COVID-19 patients with cardiac injury can determine potential risk factors or unique clinical manifestations, which will help early diagnosis of this disease and improve patient outcome.

## Methods

### Search strategy and selection criteria

We performed a comprehensive search of literature published between 1 December 2019 and 2 May 2020 on Pubmed, Embase, EuropePMC, medRxiv (https://www.medrxiv.org), SSRN (https://www.ssrn.com) and CNKI databases using the combination of the following key words: ‘COVID-19’ or ‘SARS-CoV-2’ or ‘Corona Virus Disease 2019’ or ‘cardiac injury’ or ‘myocardial damage’ or ‘heart disease’ or ‘cardiac troponin’ or ‘severity’ or ‘mortality’ or ‘ICU'without the limitation on language. After preliminary screening the literature related to the research content of this paper according to their title or abstract, we gave a thorough read of the full text and selected eligible ones to our meta-analysis. In addition, we also reviewed the references, similar documents and cited documents of the included articles to ensure the more comprehensive and accurate results.

The inclusion criteria were as follows: (1) patients included in each study were all diagnosed with COVID-19 infection and were divided into cardiac injury group and non-cardiac injury group; (2) cardiac injury was diagnosed on admission; (3) the following indicators of cardiac injury and non-injury patients were counted: age, gender, abnormal laboratory indicators, the number of patients with comorbidities such as hypertension and diabetes, with clinical complications such as AKI, ARDS and arrhythmia, with the needs for mechanical ventilation, ICU transfer and with death; (4) the sample size >20 (5) studies if they had a cohort or case−control designs. Cardiac injury was defined consistent with selected studies as hypersensitive troponin or troponin greater than the 99th-percentile upper reference limit, as per the manufacturer's indications.

The criteria for exclusion were as follows: (1) the same patients were enrolled in different studies; (2) individual case reports, reviews and editorials; (3) myocardial injury is not defined as a reference value for hypersensitive troponin or troponin above the upper 99th percentile; (4) does not meet the criteria for the heart injury grouping. Based on the above inclusion criteria, we reviewed the abstracts of 3506 studies and selected 17 eligible studies for further analysis.

### Data collection and extraction

**Fig. 2.**
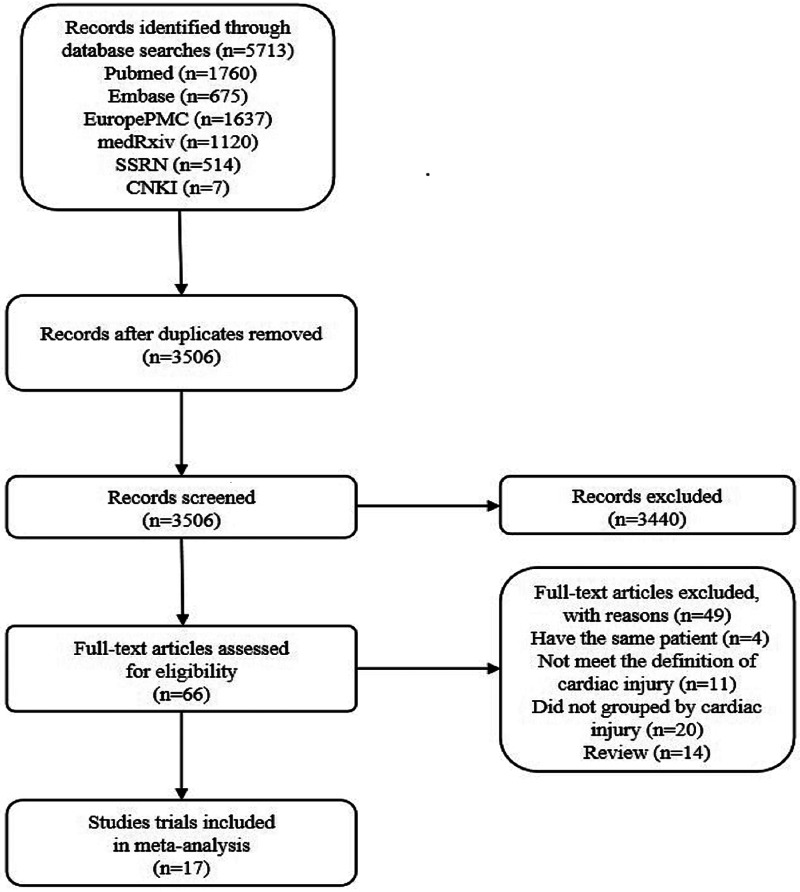
Literature search and selection process.

According to the search criteria, 2207 duplicate articles from 5713 entries were removed, and then 3440 studies were deleted after reading the titles and abstracts. After reading 66 articles, we excluded 14 reviews, 4 studies carried out with the same patients, 11 publications which did not meet the definition of cardiac injury and 20 posts that are not grouped by cardiac injury. Finally, the two authors Zeng and Wang of this study independently selected 17 eligible studies for further analyses (Fig. 2) [6, 8–23]. Later, Cai and Sun independently reviewed the abstracts or contents of 3506 studies and selected 17 eligible studies to take further analyses. The third evaluator was involved in the screening process to check for any ambiguity. Then we used the Microsoft Excel database to record the following variables from the included trials: first author, year of publication, study location, study design and relevant information mentioned in the third point of the inclusion criteria (Table 1).

**Table 1.** Characteristics and quality of the included studies

Study	Year (y)	City, Country	Number of patients (*n*) Cardiac injury		Age（years） Non-injury	Study design	Quality	Diagnostic criteria^a^
Guo *et al*. [12]	2020	Wuhan, China	52	135	44–73	Retrospective study	7	4
Liu *et al*. [24]	2020	Guangzhou, China	15	276	34–62	Retrospective study	7	3
Lala *et al*. [15]	2020	New York, America	530	1751	18–100	Retrospective study	7	3
Shi *et al*. [25]	2020	Wuhan, China	82	334	21–95	Retrospective study	7	1
Zhang *et al*. [16]	2020	Wuhan, China	13	35	57–84	Retrospective Study	8	1
Li *et al*. [18]	2020	Wuhan, China	13	88	39–76	Retrospective study	6	1
Zhou *et al*. [26]	2020	Wuhan, China	24	121	41–72	Retrospective study	7	1
Luo *et al*. [27]	2020	Wuhan, China	65	239	33–76	Retrospective study	7	1
Chen *et al*. [28]	2020	Wuhan, China	83	120	39–78	Retrospective study	7	1
Deng *et al*. [29]	2020	Wuhan, China	65	109	59–77	Retrospective study	6	1
Hu *et al*. [30]	2020	Wuhan, China	68	255	8–109	Retrospective study	7	1
Wang *et al*. [7]	2020	Wuhan, China	10	128	36–75	Retrospective study	7	3
Li *et al*. [31]	2020	Wuhan, China	119	429	43–75	Retrospective study	8	1
Du *et al*. [8]	2020	Wuhan, China	52	57	60–82	Retrospective study	7	3
He *et al*. [4]	2020	Wuhan, China	96	208	49–79	Retrospective study	8	1
Xu *et al*. [17]	2020	Sichuan, China	6	47	28–69	Retrospective study	7	4
Wei *et al*. [5]	2020	Sichuan, China	16	85	27–69	Retrospective study	8	2

a Diagnostic criteria of cardiac injury: (1) serum levels of hs-TnI were above the 99th percentile upper reference limit (0.03 ng/ml); (2) Serum levels of hs-TnT were above the 99th percentile upper reference limit (0.014 ng/ml); (3) serum levels of TnI were above the 99th percentile upper reference limit (0.03 ng/ml); (4) serum levels of TnT were above the 99th percentile upper reference limit (0.03 ng/ml).

### Quality assessment and publication bias

We used the Newcastle-Ottawa Scale (NOS) to assess the quality of the original study. At the same time, intuitive visual judgment of the symmetry and completeness of the funnel chart was carried out for qualitatively evaluating the publication bias of the included studies, while Egger's and Begg's tests were for quantitative evaluation. *P* < 0.05 represented existing publication bias.

### Statistical analysis

We applied STATA 16.0 for all meta-analysis. For dichotomised variables, we utilised Mantel−Haenszel formula for statistics and calculated RR and 95% CI; while for continuous variables, data showed as median and interquartile ranges were first converted to mean ± s.d. according to the algorithms provided by Luo *et al*. [26] and Wan *et al*. [32], and then calculated SMD and 95% CI using the inverse variance method. Heterogeneity assessment between studies was achieved with the help of the *I*^2^ test. If *I*^2^⩽50%, the results were homogeneous and a fixed-effect model could be used; Otherwise, the results were heterogeneous so a random-effect model was used. As for these heterogeneous studies, we further conducted sensitivity analysis to explore the sources of heterogeneity. *P* < 0.05 was considered statistically significant.

## Results

### Study selection and publication bias

**Fig. 3.**
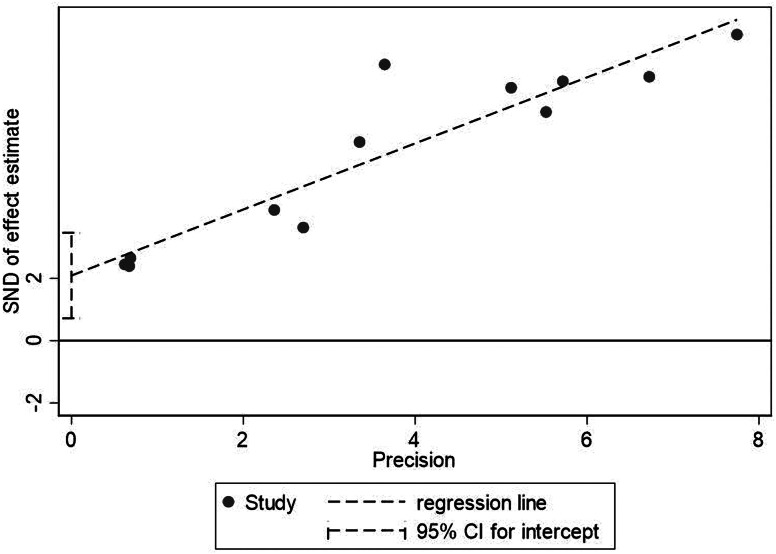
Egger's test for death between patients with cardiac injury and non-cardiac injury.

The sample size of the subjects ranged from 48 to 2281, and all studies were retrospectively analysed. In the included studies, 5 articles recorded symptoms [8, 12, 18, 19, 21], 6 posts included the laboratory index [6, 8, 12, 14, 18, 19], 6 articles recorded the complications [6, 8, 12, 14, 18, 21], 6 articles recorded the number of patients who needed mechanical ventilation or immunoglobulin therapy [6, 8, 12, 18, 21, 33], 7 articles recorded the patients who transferred to ICU [11–13, 15, 19–21], 7 studies contained comorbidities [6, 8, 12, 14, 18, 19, 21], 7 articles recorded cardiac injury/non-cardiac injury patients age and sex [6, 8, 12, 14, 19, 21, 24], 3 articles with smoking [6, 19, 21] and 12 articles recorded the situation of death [6, 8–10, 12, 17–19, 21–24] (Fig. 1). The basic information and quality scores of the studies are shown in Table 1. The NOS scores of all studies are ⩾6 points. As for the publication bias, due to the fact that (1) we do not know whether the author selectively weakened the effect of negative results in statistics to emphasise the role of positive results; (2) we may not include all the studies and most of the indicators contain less than 10 studies in the result analysis, or there are small sample studies; (3) partial results have a large heterogeneity, thus, it is not meaningful to test for publication bias for most of the results except for the death results including 12 studies. However, the value of Egger's test for the death result is 0.007, suggesting that publication bias exists. Large heterogeneity of the result and the small sample size of the individual studies included may account for it (Fig. 3).

### Age and gender

**Fig. 4.**
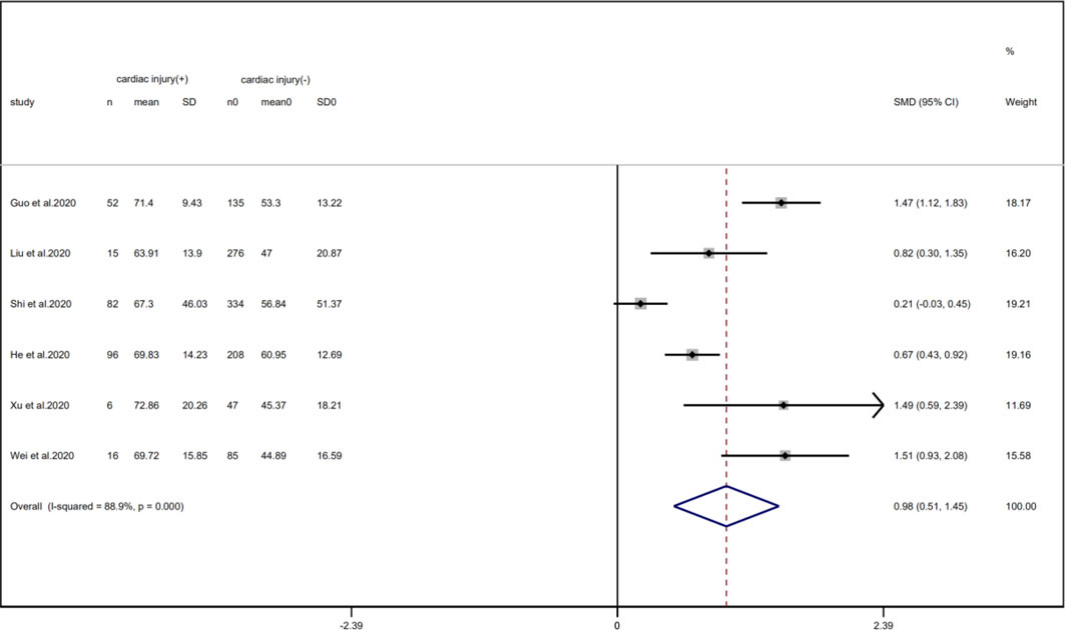
Forest plot of age difference between patients with cardiac injury and non-cardiac injury (CI, confidence interval; SMD, standard mean difference; *P* = 0.000 *I*^2^ = 88.9%).

**Fig. 5.**
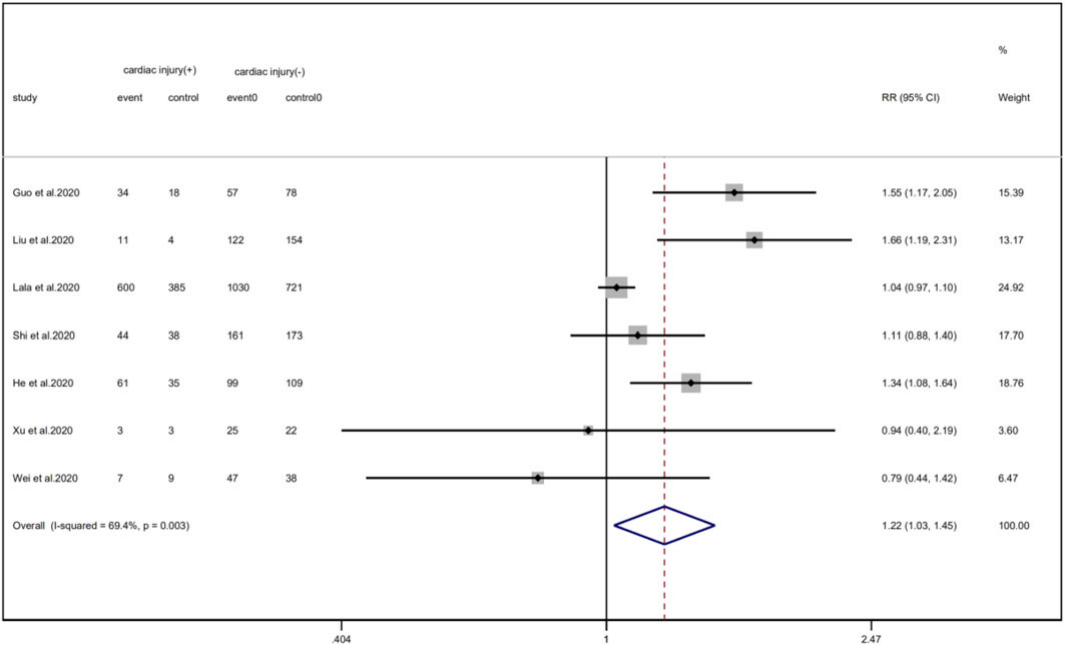
Forest plot of differences in the number of males between patients with cardiac injury and non-cardiac injury. (CI, confidence interval; RR, risk ratio; *P* = 0.003 *I*^2^ = 69.4%).

A total of 6 studies with 1352 patients indicated that the average age of the cardiac injury group ranged from 64 to 73 years, while the non-cardiac injury group was 45–61 years. The cardiac injury group was older than the non-injury group (SMD = 0.98, 95% CI 0.51–1.45, *I*^2^ = 88.9%, *P* = 0.000) (Fig. 4). Because of the large heterogeneity, we adopted the random effect model and conducted sensitivity analysis. We found that the conclusion remained unchanged but the source of heterogeneity was not clear. In addition, we observed a greater proportion of males in the cardiac injury group (RR = 1.22, 95% CI 1.03–1.45, *I*^2^ = 69.4%, *P* = 0.024) (Fig. 5). For this condition, concerning that the difference of crowd and cardiac-injured detection reagent between the study of Lala and other studies, the study of Lala might be considered as a possible source of heterogeneity in gender studies according to the sensitivity analysis (*I*^2^ dropped from 69.4% to 44.1% after deleting the study of Lala)

### Comorbidities

**Fig. 6.**
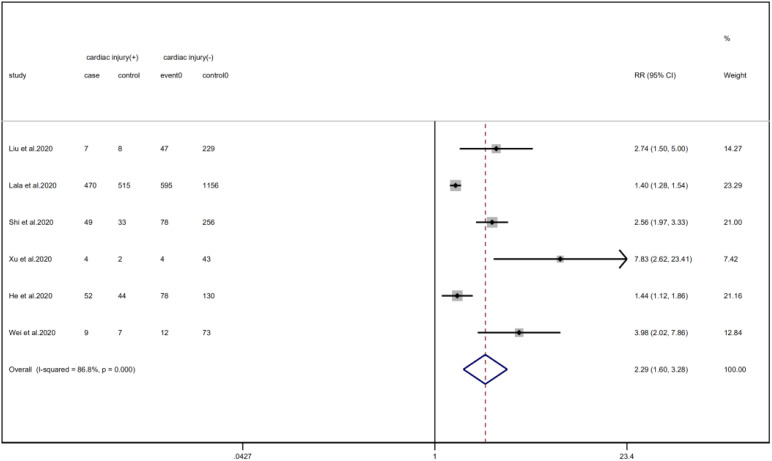
Forest plots showing differences of hypertension between patients with cardiac injury and non-cardiac injury. (CI, confidence interval; RR, risk ratio; *P* = 0.000 *I*^2^ = 86.8%).

**Fig. 7.**
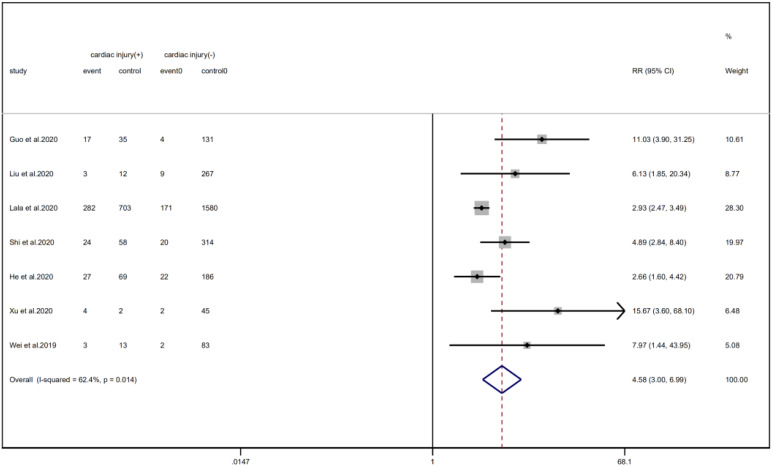
Forest plots showing differences of CHD between patients with cardiac injury and non-cardiac injury. (CI, confidence interval; RR, risk ratio; *P* = 0.014 *I*^2^ = 62.4%).

**Fig. 8.**
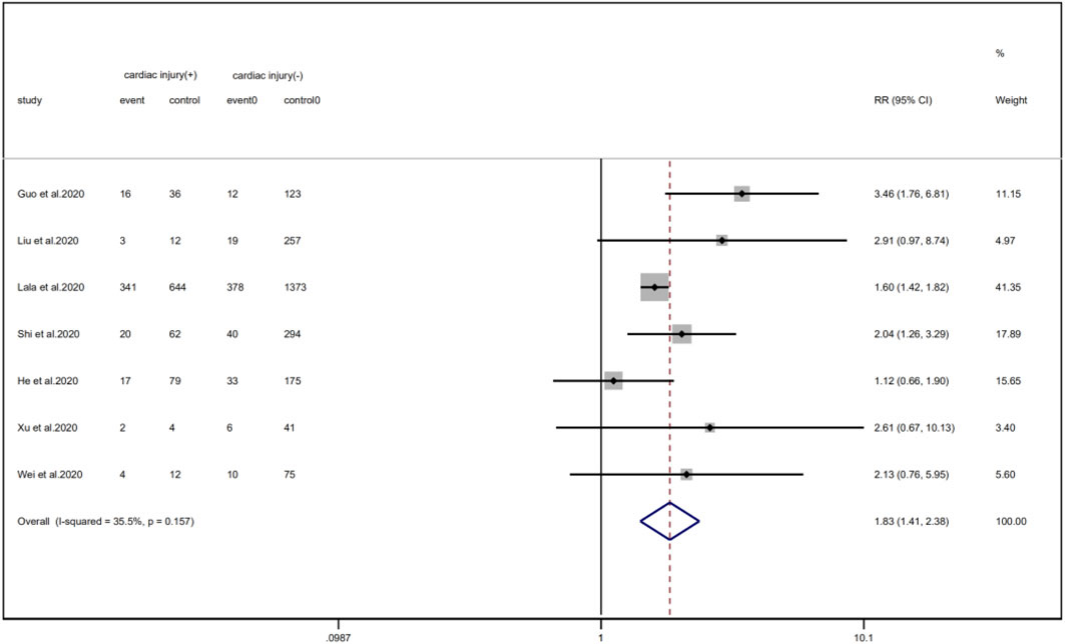
Forest plots showing differences of diabetes between patients with cardiac injury and non-cardiac injury. (CI, confidence interval; RR, risk ratio; *P* = 0.157 *I*^2^ = 35.5%).

**Fig. 9.**
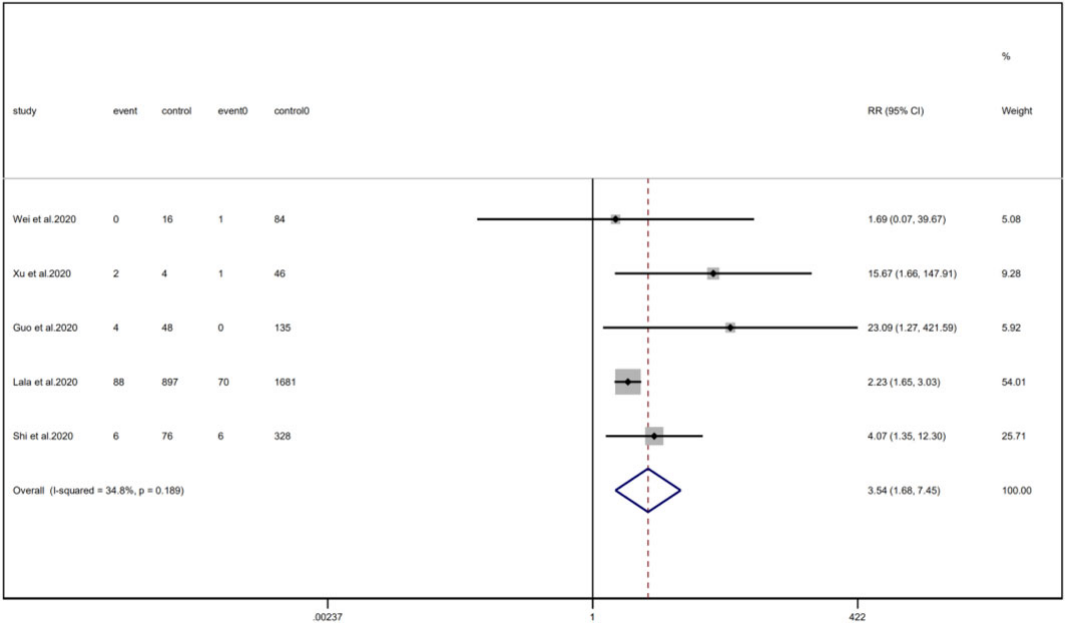
Forest plots showing differences of COPD between patients with cardiac injury and non-cardiac injury. (CI, confidence interval; RR, risk ratio; *P* = 0.189 *I*^2^ = 34.8%).

We analysed the proportion of underlying diseases in the cardiac injury *vs.* non-injury group. The results showed that the cardiac injury group was more likely to suffer from hypertension (RR = 2.29, 95% CI 1.60–3.28, *I*^2^ = 86.8%, *P* = 0.000), diabetes (RR = 1.83, 95% CI 1.41–2.38, *I*^2^ = 35.5%, *P* = 0.000), CHD (RR = 4.58, 95% CI 3.00–6.99, *I*^2^ = 62.4%, *P* = 0.000) and COPD (RR = 3.54, 95% CI 1.68–7.45, *I*^2^ = 34.8%, *P* = 0.001). Sensitivity analysis was performed on the results of hypertension and CHD due to the large heterogeneity. Though the conclusion was still unchanged after excluding each study, we noted that, as mentioned above, studies included in these two results used different cardiac-injured detection reagent, which might be the underlying source of heterogeneity (Figs. 6–9).

### Complications

**Fig. 10.**
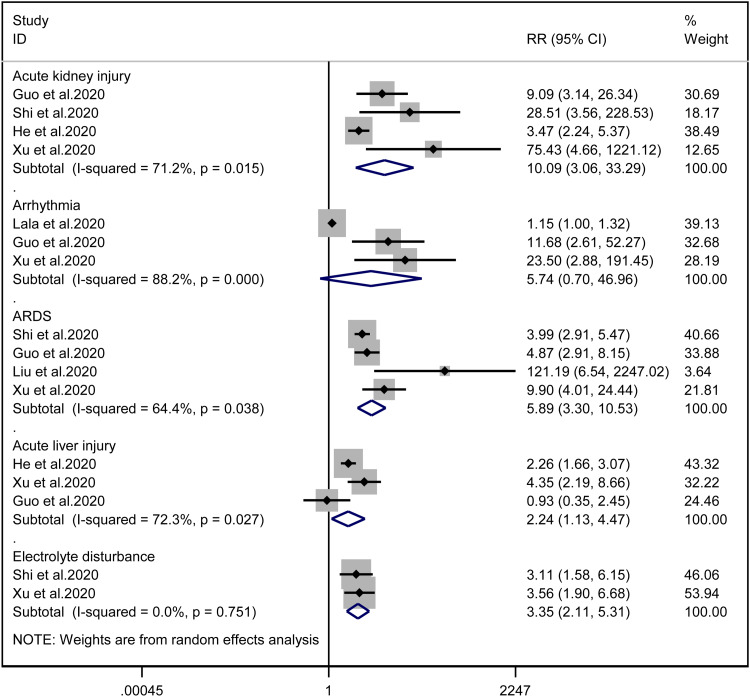
Forest plots showing differences of complication between patients with cardiac injury and non-cardiac injury. (CI, confidence interval; RR, risk ratio; *P* = 0.751 *I*^2^ = 0%).

Patients with cardiac injury are more likely to have complications such as AKI (RR = 10.09, 95% CI 3.06–33.29, *I*^2^ = 71.2%, *P* = 0.000), ARDS (RR = 5.89, 95% CI 3.30–10.53, *I*^2^ = 64.4%, *P* = 0.000), AHI (RR = 2.24, 95% CI 1.13–4.47, *I*^2^ = 72.3%, *P* = 0.022) and electrolyte disturbance (RR = 3.35, 95% CI 2.11–5.31, *I*^2^ = 0.0%). And we found that on the aspect of AKI, the study of He *et al*. may be a source of heterogeneity (*I*^2^ dropped from 71.2% to  20.3% after deleting the study of He *et al*.). However, the risk of arrhythmia between two groups did not reach statistically significant difference (RR = 5.74, 95% CI 0.70–46.96, *I*^2^ = 88.2%, *P* = 0.103). When we removed any of the studies, we would get the opposite results (Fig. 10).

### Disease outcome

**Fig. 11.**
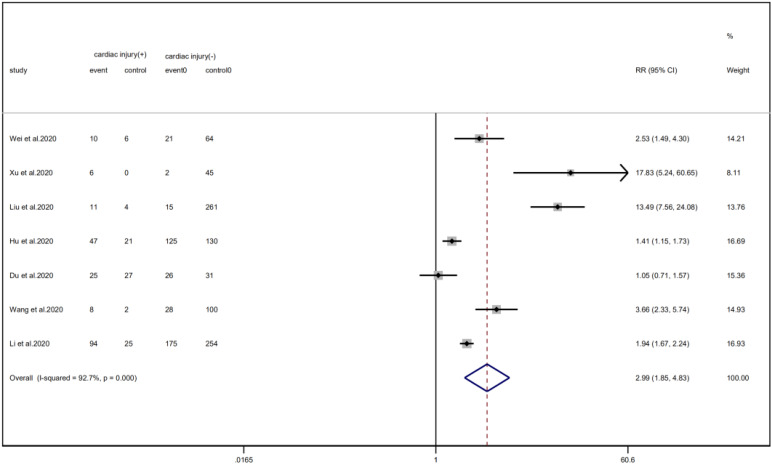
Forest plot of differences in ICU admissions between patients with cardiac injury and non-cardiac injury. (CI, confidence interval; RR, risk ratio; *P* = 0.000 *I*^2^ = 92.7%).

**Fig. 12.**
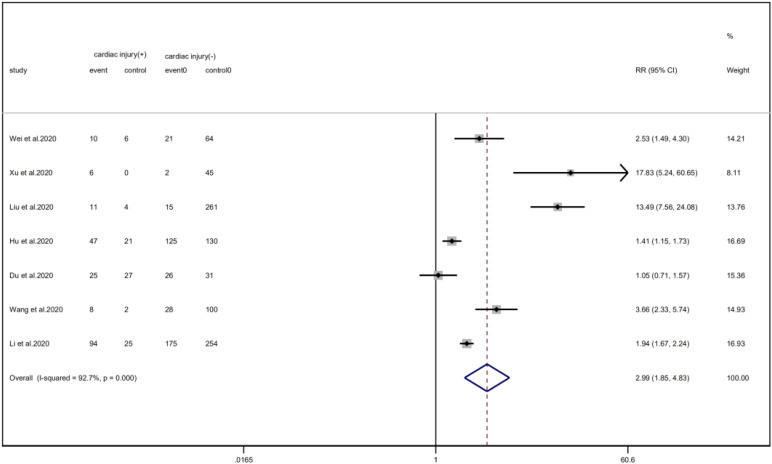
Forest plot of differences in the number of death between patients with cardiac injury and non-cardiac injury. (CI, confidence interval; RR, risk ratio; *P* = 0.004 *I*^2^ = 60.0%).

Cardiac injury patients have a higher risk of being transferred to the ICU (RR = 2.99, 95% CI 1.85–4.83, *I*^2^ = 92.8%, *P* = 0.000) (Fig. 11) and face death (RR = 4.89, 95% CI 3.84–6.22, *I*^2^ = 60.0%, *P* = 0.000) (Fig. 12) than patients without cardiac injury. Sensitivity analysis showed that the results were stable.

### Laboratory findings

Compared with non-cardiac injury group, indices indicated below were increased in cardiac injury patients, including PCT (SMD = 1.06, 95% CI 0.80–1.32, *I*^2^ = 81.3%, *P* = 0.000), CRP (SMD = 0.92, 95% CI 0.42–1.43, *I*^2^ = 95.6%, *P* = 0.000), D-dimer (SMD = 0.89, 95% CI 0.66–1.12, *I*^2^ = 69.3%, *P* = 0.000), NT-pro BNP (SMD = 1.75, 95% CI 1.22–2.28, *I*^2^ = 90.6%, *P* = 0.000), and serum creatinine (SMD = 0.97, 95% CI 0.74–1.21, *I*^2^ = 77.8%, *P* = 0.000). However, in cardiac injury patients, the count of lymphocytes (SMD = −0.71, 95% CI 0.93–0.49, *I*^2^ = 52.7%, *P* = 0.000) and platelets (SMD = 0.62, 95% CI 0.79–0.46, *I*^2^ = 0.0%, *P* = 0.000) was decreased. When we removed the study of Lala *et al*., the *I*^2^ of other remaining studies in PCT was significantly reduced (<50%). Therefore, the study by Lala is the main source of heterogeneity and possible reasons have been stated above. Interestingly, no significant differences were observed in the plasma concentration of Na^+^ (SMD = 0.13, 95% CI −0.33 to 0.60, *I*^2^ = 92.6%, *P* = 0.573) and K^+^(SMD = −0.05, 95% CI −0.43 to 0.32, *I*^2^ = 71.6%, *P* = 0.778) between two groups (Fig. 8, 9, 13–21).

**Fig. 13.**
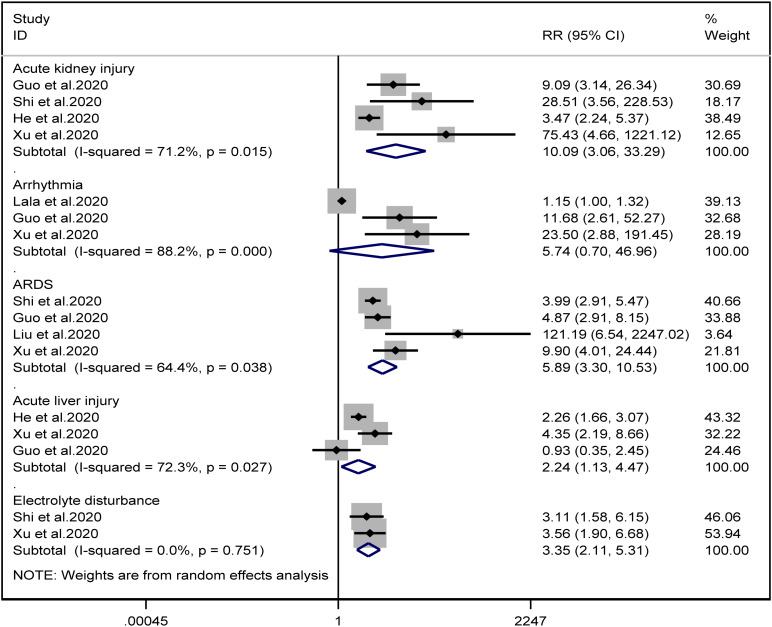
Forest plots showing differences of complication between patients with cardiac injury and non-cardiac injury. (CI, confidence interval; RR, risk ratio; *P* = 0.751 *I*^2^ = 0%).

**Fig. 14.**
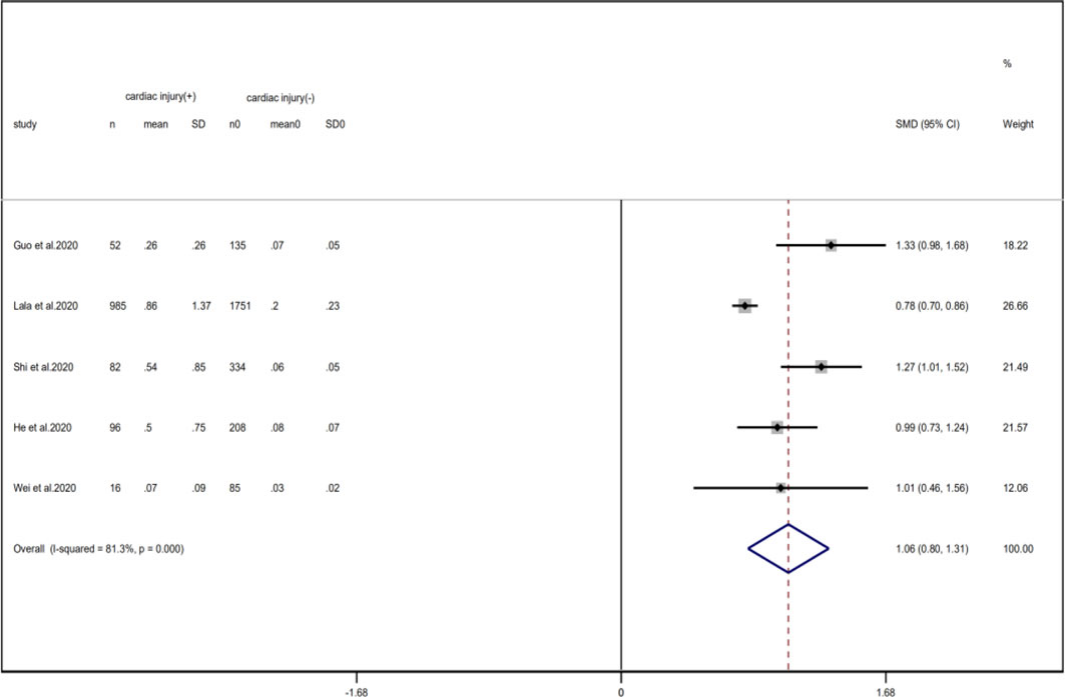
Forest plots showing differences of PCT between patients with cardiac injury and non-cardiac injury. (CI, confidence interval; RR, risk ratio; *P* = 0.000 *I*^2^ = 81.3%).

**Fig. 15.**
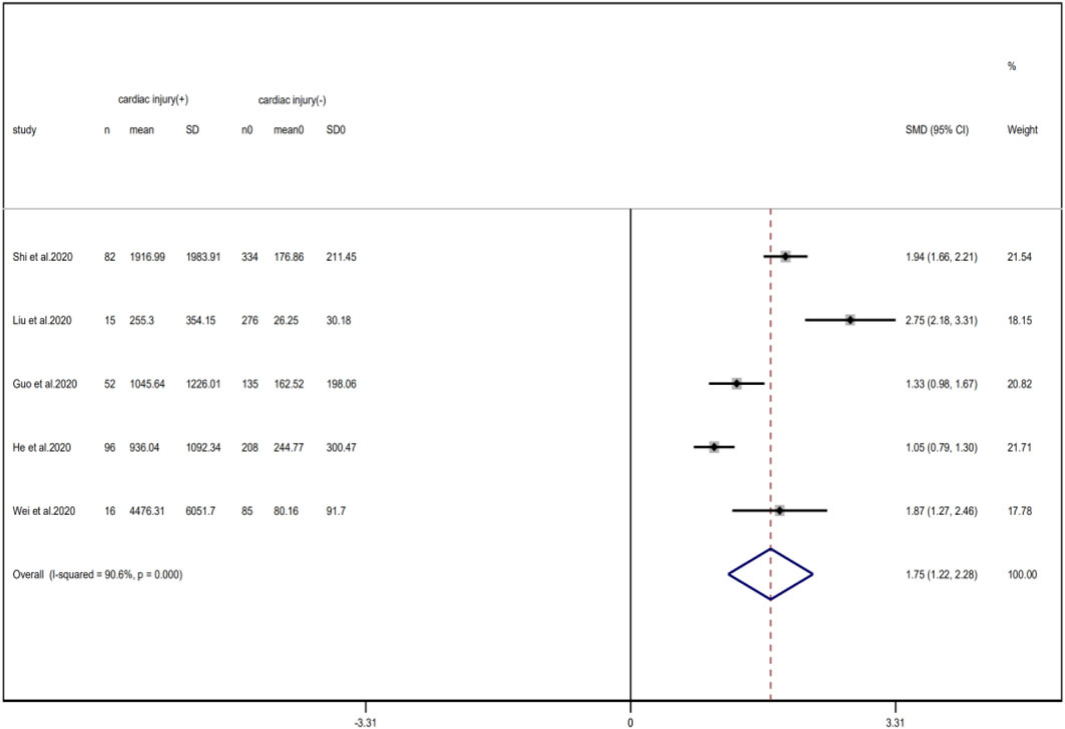
Forest plots showing differences of NT-proBNP between patients with cardiac injury and non-cardiac injury. (CI, confidence interval; RR, risk ratio; *P* = 0.000 *I*^2^ = 90.6%).

**Fig. 16.**
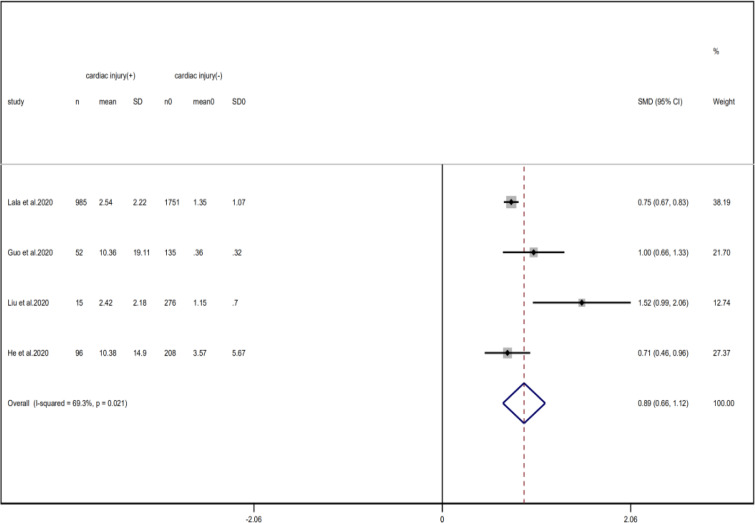
Forest plots showing differences of D-dimer between patients with cardiac injury and non-cardiac injury. (CI, confidence interval; RR, risk ratio; *P* = 0.021 *I*^2^ = 89.3%).

**Fig. 17.**
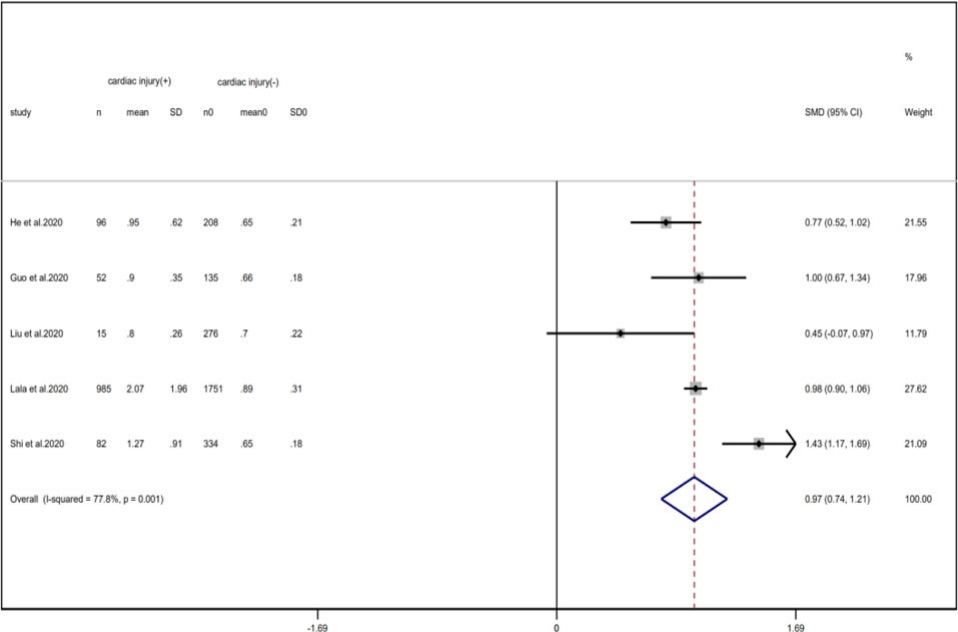
Forest plots showing differences of creatinine between patients with cardiac injury and non-cardiac injury. (CI, confidence interval; RR, risk ratio; *P* = 0.001 *I*^2^ = 77.8%).

**Fig. 18.**
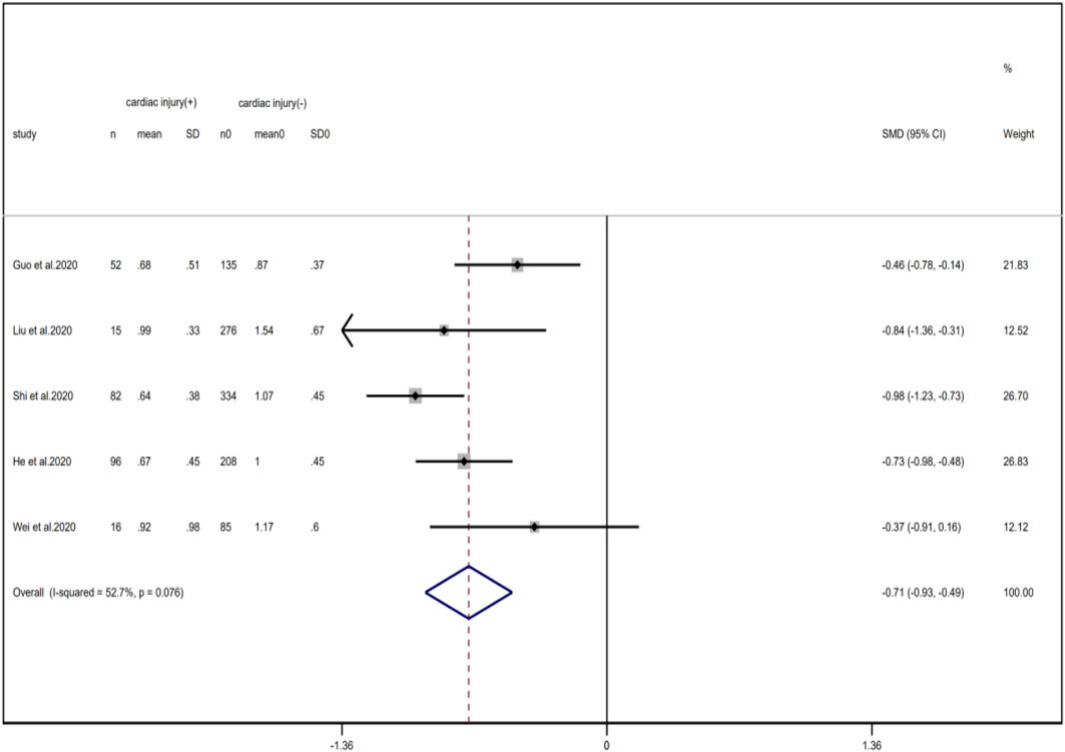
Forest plots showing differences of lymphocyte counts between patients with cardiac injury and non-cardiac injury. (CI, confidence interval; RR, risk ratio; *P* = 0.076 *I*^2^ = 52.7%).

**Fig. 19.**
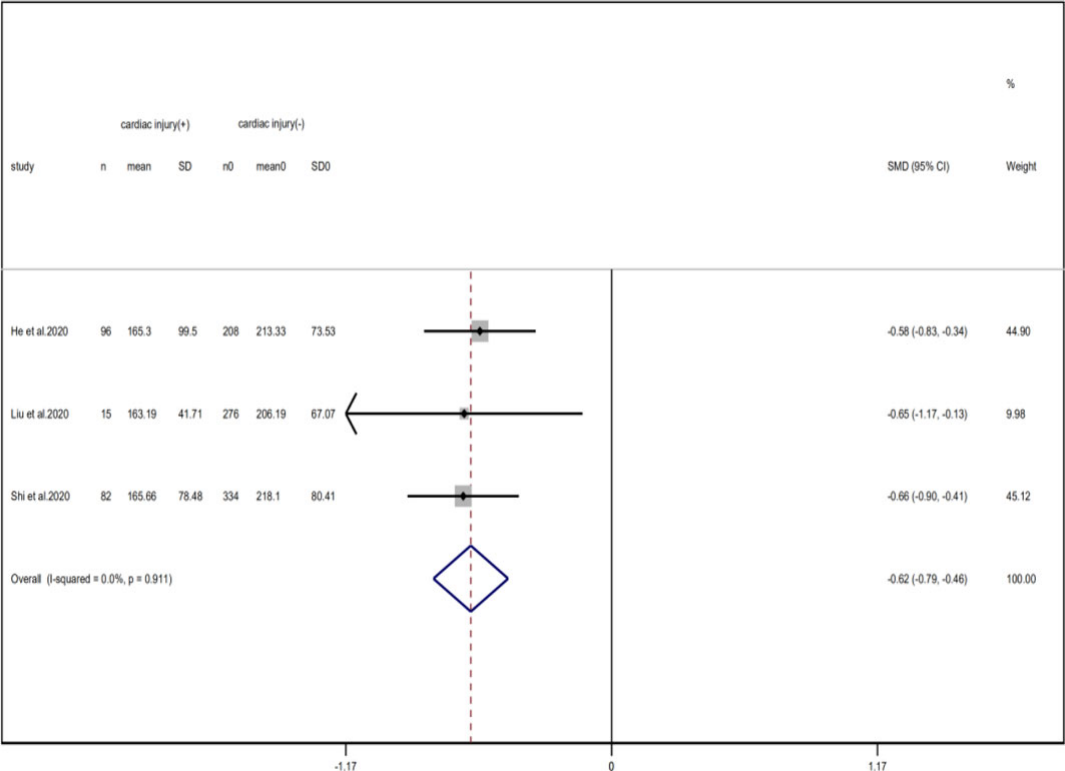
Forest plots showing differences of platelet counts between patients with cardiac injury and non-cardiac injury. (CI, confidence interval; RR, risk ratio; *P* = 0.911 *I*^2^ = 0%).

**Fig. 20.**
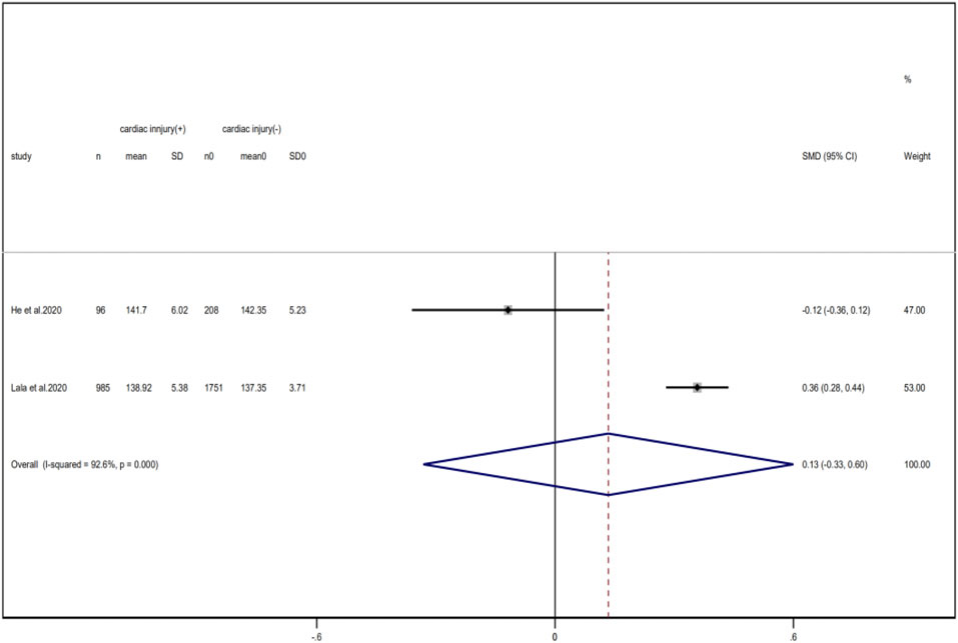
Forest plots showing differences of Na^+^ between patients with cardiac injury and non-cardiac injury. (CI, confidence interval; RR, risk ratio; *P* = 0.000 *I*^2^ = 92.6%).

**Fig. 21.**
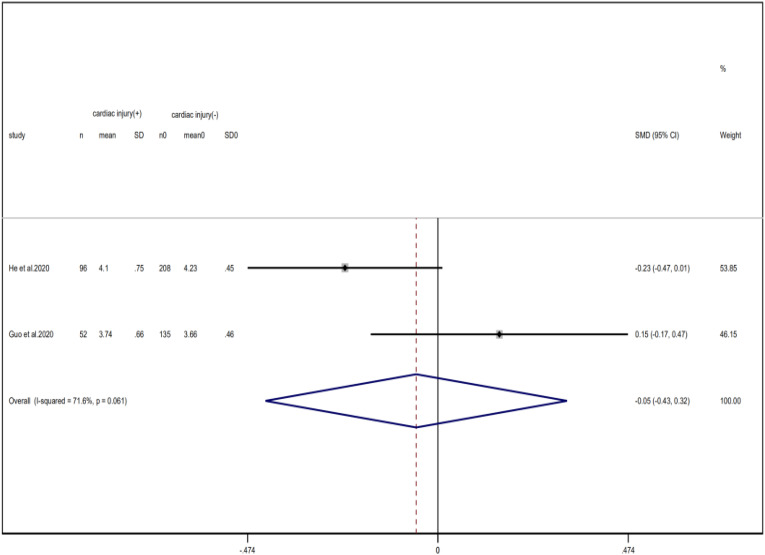
Forest plots showing differences of K^+^ between patients with cardiac injury and non-cardiac injury. (CI, confidence interval; RR, risk ratio; *P* = 0.061 *I*^2^ = 71.6%).

### Smoking

Compared with the control group, the number of smoking patients showed an increase in cardiac injury group but did not reach statistical difference (RR = 3.43, 95% CI 0.45–26.24, *I*^2^ = 79.2%, *P* = 0.234) (Fig. 5).

### Symptoms

Our analysis showed that, between groups of patients with or without cardiac injury, there was no significant difference in the incidence of chest pain (RR = 2.53, 95% CI 0.38–16.65, *I*^2^ = 77.2%, *P* = 0.334), chest discomfort (RR = 1.18, 95% CI 0.86–1.62, *I*^2^ = 0.0%, *P* = 0.304) and palpitation (RR = 0.79, 95% CI 0.14–4.45, *I*^2^ = 0.0%, *P* = 0.793). Moreover, although the total effect value of dyspnoea was slightly higher than the invalid value (RR = 1.48, 95% CI 1.05–2.08, *I*^2^ = 33.9%, *P* = 0.025), the opposite conclusion was obtained after excluding the study of He *et al*. (Fig. 7). This suggests that the results regarding dyspnoea analysis were unstable.

### Treatments

**Fig. 22.**
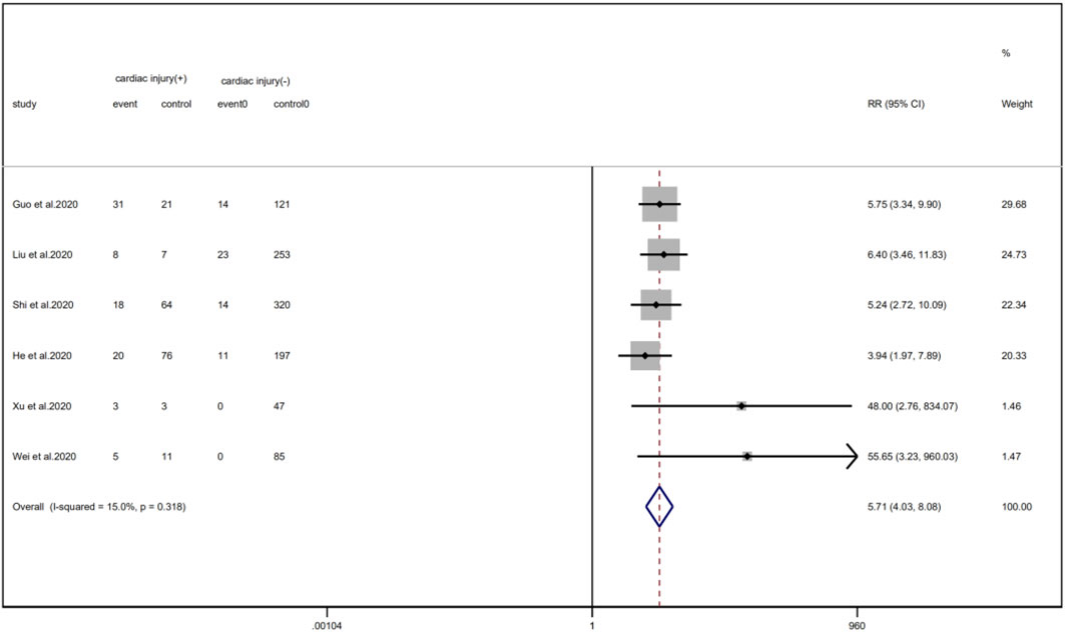
Forest plots showing immunoglobulin therapy between patients with cardiac injury and non-cardiac injury. (CI, confidence interval; RR, risk ratio; *P* = 0.000 *I*^2^ = 91.9%).

Meta-analysis showed that patients with cardiac injury required more mechanical ventilation than non-cardiac injury patients (RR = 5.71, 95% CI 4.04–8.08, *I*^2^ = 15.0%, *P* = 0.000) (Fig. 22).

## Discussion

In this systematic review and meta-analysis, we found that cardiac injury complicated COVID-19 infection more likely occurred in older male patients. COVID-19 patients with cardiac injury are susceptible to complications and usually have abnormal results of laboratory tests, leading to worse outcomes. Unexpectedly, cardiac injury complicated COVID-19 infection did not significantly induce chest distress, chest pain, palpitations or arrhythmias. We also identified that comorbidities and the inflammation index were independent risk factors for cardiac injury that occurred in COVID-19 infection.

Age is an independent risk factor for many diseases, and our results connected it with the cardiac injury. When being infected by the SARS-CoV-2, myocardial cells in older people are more inclined to get out of balance due to the down-regulated cell homeostasis, as a result, cardiac injury occurs. Besides, the ageing of the immune system can cause the loss of coordination among CD4^+^T cells and significantly reduce the immune function [28], which leads to the massive replication of the virus and further aggravating the damage to the heart. Recently, a meta-analysis of Zou *et al*. [33] has also revealed that age shows a tight correlation with the development of cardiac injury, consistent with our results, so age may be an indicator of COVID-19 complicated cardiac injury.

Male gender is another risk factor for cardiac injury in COVID-19 infection. Although this result did not manifest statistical significance in a pre-print study of 2736 patients by Lala *et al*. [14], our pooled analysis of seven studies reported that cardiac injury was more common in men. This phenomenon might be related to the viral infection mechanism. SARS-CoV-2 uses ACE2 as a medium to attack target cells. And lately, it has been reported that women have a higher ACE2 shedding rate and are more likely to produce SARS-CoV-2 IgG antibodies in the early stages of the disease [31, 34], therefore these patients are more conducive to the initiation of off-target and protective effects when the virus attacks the heart.

It seems a consensus that cardiac injury is closely related to comorbidities. The probability of cardiac injury in patients with basic diseases is 47.3%, while in patients without underlying diseases, it dropped to 27.1%. Statistically, hypertension (49.3%), diabetes (32.1%), CHD (28.8%) and COPD (8.8%) are common basic diseases in cardiac-injured patients, which are in line with the studies by Xu *et al*. [35]. Another multivariable logistic regression analysis also deemed CHD as a predictor of cardiac injury [36]. As for the possible mechanism, it may ascribe to the imbalance of oxygen supply in the body when viruses infect and then chronic underlying diseases lose their original stability. In anoxic environment, massive accumulation of lactic acid lead to abnormal coronary systole, increasing the risk of non-ischaemic myocardial injury and type II myocardial infarction [29].

The majority of COVID-19 patients shows increased inflammatory indicators. Viral infection not only activates the inherent and adaptive immune response, resulting in the release of a large number of cytokines and chemokines, but also amplifies the synergistic effect of AngII on the pro-inflammatory pathway by down-regulating ACE2 [24]. Consistent with previous studies by Shi [8], we found that the inflammation index of patients with cardiac injury was significantly increased compared with those without injury. This phenomenon may be related to the secondary immune response of the host, the regulation of T lymphocyte depletion, or the imbalance of NF-kB/IRF3-IRF7 pathway [24, 25]. Considering that inflammation is closely related to infection and the severity of the disease [37], so patients with cardiac injury may be more susceptible to infection, leading to disease progression and poor prognosis. Similarly, platelet and lymphocyte counts also reflect the degree of infection and inflammation control in the body. Early, pro-inflammatory states in COVID-19 infection induce platelet proliferation and lymphocyte migration. In the later stages, both of the content are greatly reduced because of direct destruction by the virus or secondary DIC and thrombosis, contrasting to the finding that the ratio between platelet and lymphocyte count is always proportional to the time at hospital [30]. In this study, the platelet and lymphocyte counts in the patients with cardiac injury were lower than those in the patients without injury, but the ratio of two indicators was significantly higher. The COVID-19 patients with cardiac injury had a more serious disease and a greater risk for secondary adverse thrombotic events. Other laboratory indices need to be monitored for patients with cardiac injury include creatinine and NT-pro BNP. Creatinine is an important index reflecting renal function, and is also one of the endogenous substances causing cardiac injury. Therefore, its increase may indicate secondary renal insufficiency in patients. NT-pro BNP is a specific indicator of heart failure and recently Wei *et al*. pointed out that it was associated with the progression of COVID-19 infection [19]. Another study found that it was higher than the normal value in the middle of hospitalisation and reached a peak immediately before death [4]. Likewise, our analysis indicated its increase in cardiac-injured patients. Thereby, early, real-time and dynamic monitoring of these laboratory indicators in patients with cardiac injury will help to judge disease progression and guide treatment.

ARDS, AKI and AHI are common clinical complications in COVID-19 patients. A recent univariate analysis included these complications in risk factors for cardiac involvement [35] and meanwhile, Mohamad *et al*. found that the incidence of AKI and ARDS was positively correlated with the degree of cardiac injury [38]. Our analysis also elucidated that cardiac-injured patients were more easily concurrent with these diseases, owing to the cytokine storms, blood hypercoagulability, blocked circulation and preferential blood supply to important organs. As the diseases progressed, hypoxia, acidic metabolite accumulation and decreased renal filtration function further mediated ionic balance disorders. Therefore, physicians should pay attention to the relevant symptoms and laboratory test results and intervene these complications as soon as possible. Surprisingly, we did not find an increase in risk for arrhythmia in cardiac-injured patients. This conclusion may hold truth, since the cause for arrhythmia in COVID-19 patients is not only due to myocardial damage but also fever, electrolyte disturbance, antiviral drug and hypoxaemia [3]. However, we need larger sample size to confirm this conclusion since the sensitivity analysis suggested that these results were not robust and the study of Lala *et al*. may be the source of heterogeneity (Fig. 9).

Beyond our expectation, the risks for chest discomfort, chest pain and palpitation are not increased in cardiac-injured patients, possibly because pneumonia or other respiratory diseases can also result in these symptoms. Liu *et al*. [18] reported the same results, hence, using these symptoms as risk factors to predict the occurrence of cardiac injury is not reliable in COVID-19 patients.

Smoking is a risk factor for a variety of cardiovascular diseases. Zhang *et al*. recently published an article that nicotine induced myocardial injury by enhancing autophagy signalling pathway and ROS expression [39]. Surprisingly, smoking did not increase the risk of myocardial damage in the study. This may be due to the relatively small sample size and large heterogeneity included in this study, so further research is needed.

Patients with cardiac injury seem to have a poor prognosis and our conclusion is consistent with this description by Santoso *et al*. [5]. The reason leading to death in COVID-19 patients with cardiac injury is not yet clear, possibly due to cytokine storm syndrome or fulminant myocarditis [7, 27]. Presently, no virus particles have been found in cardiac myocytes according to autopsy reports, so large-scale analysis of case reports and autopsy reports is necessary.

In brief, the above sections discuss the relationship between various influencing factors and cardiac injury, however, the research studies we included could not represent all the studies and the publication bias of some articles may affect the authenticity of the results. Particularly, when published articles tend to focus more on positive results other than negative results to some extent, it may trigger a inductive effect on the results of this meta-analysis. Besides, considering that this analysis also includes small sample size studies and majority of the studies are from China, neglecting the impact of ethnic and regional differences, and that the differences on the design schemes and inclusion criteria between studies can also change the quality of including articles, this fact may have a certain influence on the reliability of our results. So, looking forward to more comprehensive research in the future is indispensable.

### Limitation

This study rigorously analyses the scientific publications from different regions of China and the United States, but there are some limitations that need to be addressed in the future. First, the studies included in this paper are mostly retrospective studies, lacking randomised-controlled studies with optimised design, and some of the analyses are only based on two studies, so expanding the patient sample for further systematic analysis is needed. Second, some analyses here may have publication bias, because some of studies included here are preprinted or there are no sufficient relevant reports. Third, some analyses display greater heterogeneity and no source of heterogeneity is found after sensitive analysis. Moreover, we did not conduct subgroup analysis to discuss the source of heterogeneity due to the scarceness of relevant studies, so further study was still needed.

## Conclusion

Age, male gender, co-existing diseases and the inflammation index are risk factors for cardiac injury complicated COVID-19 infection. When a patient meets these criteria, cardiac-injured biomarkers, such as cardiac troponin and CK-MB, should be monitored. COVID-19 patients with cardiac injury are usually at higher risk for clinical complications, mechanical ventilation and death, suggesting intensive care may be required for those patients. Smoking history and clinical manifestations of common cardiovascular disease are not independent risk factors to predict cardiac injury complicated COVID-19 infection. Our study indicates that early prevention should be applied to COVID-19 patients with cardiac injury to reduce adverse outcomes.

## Highlights

1. Age, male and previous existence of hypertension, diabetes, CHD, COPD are risk factors for cardiac injury in COVID-19 patients.
2. Chest tightness, chest pain and palpitations are not reliable indicators of the cardiac injury complicated COVID-19 infection.
3. The levels of PCT, CRP, D-dimer, NT-proBNP and creatinine are increased in patients with cardiac injury; while the counts of lymphocyte and platelet are reduced in those patients. Hence, monitoring these laboratory indices can help judge disease progression.
4. COVID-19 patients with cardiac injury are usually at higher risk for clinical complications, mechanical ventilation and death, suggesting intensive care may be required for these patients.

## Data Availability

Li, Jingjie Li, 2020, ‘Clinical characteristics of Covid-19 with cardiac injury: a systematic review and meta-analysis’, https://doi.org/10.7910/DVN/URIKK4, Harvard Dataverse, DRAFT VERSION.
